# A mixed-effects two-part model for twin-data and an application on identifying important factors associated with extremely preterm children’s health disorders

**DOI:** 10.1371/journal.pone.0269630

**Published:** 2022-06-13

**Authors:** Baiming Zou, Hudson P. Santos, James G. Xenakis, Mike M. O’Shea, Rebecca C. Fry, Fei Zou

**Affiliations:** 1 Department of Biostatistics, University of North Carolina at Chapel Hill, Chapel Hill, NC, United States of America; 2 School of Nursing, University of North Carolina at Chapel Hill, Chapel Hill, NC, United States of America; 3 Department of Genetics, University of North Carolina at Chapel Hill, Chapel Hill, NC, United States of America; 4 Division of Neonatal-Perinatal Medicine, University of North Carolina at Chapel Hill, Chapel Hill, NC, United States of America; 5 Department of Environmental Sciences and Engineering, University of North Carolina at Chapel Hill, Chapel Hill, NC, United States of America; Universidade Federal do Rio Grande do Sul, BRAZIL

## Abstract

Our recent studies identifying factors significantly associated with the positive child health index (PCHI) in a mixed cohort of preterm-born singletons, twins, and triplets posed some analytic and modeling challenges. The PCHI transforms the total number of health disorders experienced (of the eleven ascertained) to a scale from 0 to 100%. While some of the children had none of the eleven health disorders (i.e., PCHI = 1), others experienced a subset or all (i.e., 0 ≤PCHI< 1). This indicates the existence of two distinct data processes—one for the healthy children, and another for those with at least one health disorder, necessitating a two-part model to accommodate both. Further, the scores for twins and triplets are potentially correlated since these children share similar genetics and early environments. The existing approach for analyzing PCHI data dichotomizes the data (i.e., number of health disorders) and uses a mixed-effects logistic or multiple logistic regression to model the binary feature of the PCHI (1 vs. < 1). To provide an alternate analytic framework, in this study we jointly model the two data processes under a mixed-effects two-part model framework that accounts for the sample correlations between and within the two data processes. The proposed method increases power to detect factors associated with disorders. Extensive numerical studies demonstrate that the proposed joint-test procedure consistently outperforms the existing method when the type I error is controlled at the same level. Our numerical studies also show that the proposed method is robust to model misspecifications and it is applicable to a set of correlated semi-continuous data.

## 1 Introduction

Approximately 10% of babies are born prematurely (< 37 weeks gestational age) worldwide every year [1]. Preterm birth is the leading cause of neonatal mortality and an important risk factor for developmental impairments including cognitive, behavioral, and social-emotional disorders [2–7]. Individuals born preterm also experience a higher risk of health disorders (e.g., asthma) and have a shorter life expectancy [8]. Our team has investigated 11 health disorders that are associated with preterm birth including bilateral blindness, hearing impairment, moderate/severe cognitive impairment, epilepsy, gross motor function impairment, attention-deficit/hyperactivity disorder, anxiety, depression, asthma, autism, and obesity (i.e., body mass index above the 95 percentile) [9]. Further, preterm birth in the U.S. is associated with billions of dollars of annual societal economic burden [10]. On the other hand, some preterm children do not display major adverse health or developmental outcomes [9, 11]. Investigating the outcomes of preterm-born children remains critical for evaluating and improving clinical care, planning long-term support and for advancing our understanding of the life-course consequences of immaturity at birth [7]. Identifying important factors that are associated with the risk and burden of such health disorders is important to both clinicians and researchers [12]. Indeed, determining important biomarkers associated with adverse health outcomes has long been recognized as a critical component in investigating disease etiologies, developing new therapeutic interventions, and accurately predicting disease progression [13, 14]. However, the identification of such biomarkers remains a challenge.

The current research is motivated by recent epidemiological studies investigating the positive child health outcomes among 10-year-old children who were born extremely preterm [9, 12]. One of our primary scientific interests in these studies was to identify important factors associated with the positive child health index (PCHI) outcome, which summates information about the presence or absence of 11 adverse health disorders at age 10 and transforms the cumulative number to a scale from 0 (the child experienced all the disorders) to 100% (no disorders), accounting for the number of non-missing responses [12]. That is, the fewer disorders the child experienced, the higher the child’s PCHI score. Two aspects of the PCHI lead to analytic and modeling challenges when investigating associations between maternal antecedents, child’s characteristics and PCHIs. The first involves the semi-continuous nature of the scale, which clearly reveals two distinct underlying data processes. While some children experienced none of these disorders (i.e., PCHI = 1), many others experienced a subset or all (i.e., PCHI < 1). That is, the PCHI, or alternatively the number of health disorder measurements, follows a mixture distribution, taking a boundary value under one condition, or a value arising from a continuous (ordinal) distribution under another. The second challenging feature of these data was that, in addition to singletons, it included twin and triplet clusters, within which PCHI measurements, or equivalently the number of health disorder experienced by each of the twins and triplets were correlated, as members of the same nuclear family share similar genetic structures and are exposed to comparable early environments. Even more challenging is the fact that these family-related cluster effects which within the two underlying data processes impact the propensity of incurring adverse health outcome and their subsequent burden, respectively, are themselves usually correlated with each other.

Previous research employed a strategy to identify important risk factors associated with the PCHI by dichotomizing the scale based on the number of disorders experienced (no any health disorder versus at least one health disorder, i.e., PCHI= 1 versus PCHI< 1) and using this binary outcome in a mixed-effects logistic regression with a random intercept to correlate children from a multiple birth [12]. Although this modeling scheme accommodates all the samples and is mathematically convenient, it can limit statistical power, since it is only capable of capturing the impact of risk factors on a binary health disorder status. However, in practice, these risk factors might also impact the disease burden, i.e., these risk factors may not only influence the propensity of developing health disorders but also impact the burden of disease (i.e., the number of health disorder experienced). More importantly, these risk factors usually impact both positively or negatively on the two data processes, i.e. in the same direction. Thus, jointly modeling the effects of these factors on the health outcomes can boost detection power. For independent samples, to jointly model this type of semi-continuous data, a two-part model can be adopted [15–20], where a logistic regression model is used to model the binary event of incurring no health disorders versus incurring at least one disorder, while a multiple linear (log-normal) regression is employed to model the association between the factors and the burden of health disorders (i.e., the nonzero data where the number of disorder experienced is converted to the proportion of the total 11 disorder, the larger the more severe). Special treatment is still required to account for the correlations among twin or triplet samples. To this end, we adopt the mixed-effects two-part modeling framework developed for longitudinal semi-continuous data [21]. Jointly, we model the correlated semi-continuous data by taking into account the sample correlations between and within the two underlying data processes. Under this framework, we derive a joint-test procedure for assessing each risk factor’s effect on the correlated semi-continuous outcome measurements.

The remainder of the paper is arranged as follows. In Section 2, we provide a detailed description of the proposed mixed-effects two-part modeling framework for the correlated semi-continuous data. We then apply the proposed joint-test procedure to identify important factors associated with the health disorders for extremely preterm children in Section 3 to demonstrate its benefits over the existing method. We conduct extensive numerical studies under different data complexity scenarios in Section 4 to explore the applicability and performance as compared with the existing method and to demonstrate the generalizability and robustness of the proposed joint-test procedure for the correlated semi-continuous data. The paper concludes with some discussions in Section 5.

## 2 Joint-test procedure under mixed-effects two-part model for correlated semi-continuous data

Before providing more detailed explication of our proposed joint-test procedure, we first introduce some notations. Let **X** represent a matrix of all the observed clinical, demographic and biomarker variables, and *Y* represent an outcome vector of interest (e.g., the health disorder score), which can be zero or a continuous positive value. In the context of PCHI, equivalently, *Y* = 1 − PCHI where zero corresponds to experiencing no any adverse health issues while a positive value represents experiencing the proportion of 11 adverse health disorders (the larger the value, the more adverse outcomes experienced). We introduce another binary variable vector, *Z*, which is a dichotomization of the adverse health status, such that *Z* = **I** (*Y* > 0) where **I** is an indicator function. Our primary scientific interest is to model Pr(*Y* > 0 | **X**) and E[*Y*|*Y* > 0, **X**)] such that we can identify risk factors that are significantly associated with the propensity of being in disease status (i.e., *Z* = 1) and the burden of disease (i.e., *Y* > 0) when in disease state.

To model the association between the observed risk factors **X** and the adverse health status *Z* for the correlated data, we adopt a mixed-effects multiple logistic regression model, while the impacts of risk factors on the burden of the adverse health outcome are modelled with a log-normal distribution as follows, although other mixed-effects (e.g., linear or Poission) models can be employed. Our models can be written as follows:

$$\begin{array}{ccc} \text{logit}(\pi_{ij}) & = & \alpha_{0}+\sum_{k=1}^{p}\alpha_{k}x_{k,ij}+u_{i} \end{array}$$ (1)

$$\begin{array}{ccc} \text{log}\left(y_{ij}\right)|y_{ij}>0 & = & \beta_{0}+\sum_{k=1}^{p}\beta_{k}x_{k,ij}+v_{i}+\epsilon_{ij} \end{array}$$ (2)

where *x*_*k*,*ij*_ is the *k*^*th*^ observed risk factor of the *j*^*th*^ subject in family *i* (*i* = 1, ⋯, *m*; *j* = 1, ⋯, *n*_*i*_), *π*_*ij*_ ≡ Pr(*z*_*ij*_ = 1|{**x**_*ij*_, *u*_*i*_}) and *ϵ*_*ij*_ ∼ *N* (0, *σ*^2^) represents a random error term. The family-specific effects on the propensity to have at least one of the health disorders, and the proportion of having all health disorders (conditional on having at least one of them) are represented by *u*_*i*_ and *v*_*i*_, respectively; to characterize the correlation between them, we assume they follow a bivariate normal distribution with mean zero as following:

$$\begin{array}{c} (\begin{array}{c} u_{i}\\ v_{i} \end{array})∼N((\begin{array}{c} 0\\ 0 \end{array})\;,\;(\begin{array}{cc} \sigma_{u}^{2} & \rho \sigma_{u}\sigma_{v}\\ \rho \sigma_{u}\sigma_{v} & \sigma_{v}^{2} \end{array})). \end{array}$$

We can write down the total likelihood function as follows:

$$\begin{array}{ccc} L & = & \prod_{i=1}^{m}\int_{u_{i}}\int_{v_{i}}\prod_{j=1}^{m_{i}}f\left(z_{ij},y_{ij}|\alpha ,\beta ,u_{i},v_{i},\sigma^{2}\right)f\left(u_{i},v_{i}|\sigma_{u}^{2},\sigma_{v}^{2},\rho \right)dv_{i}du_{i}\\ & = & \prod_{i=1}^{m}\int_{u_{i}}\int_{v_{i}}\prod_{j=1}^{m_{i}}{\left\{1-Pr\left(z_{ij}=1|\alpha ,u_{i}\right)\right\}}^{\left(1-z_{ij}\right)}{\left\{Pr\left(z_{ij}=1|\alpha ,u_{i}\right)\right\}}^{z_{ij}}\\ & & {\left[f\left(y_{ij}|\beta ,v_{i},\sigma^{2}\right)\right]}^{z_{ij}}f\left(u_{i},v_{i}|\sigma_{u}^{2},\sigma_{v}^{2},\rho \right)dv_{i}du_{i} \end{array}$$ (3)

where ***α*** = (*α*_0_, *α*_1_, ⋯, *α*_*p*_), ***β*** = (*β*_0_, *β*_1_, ⋯, *β*_*p*_), representing the risk factor effects on the propensity of developing at least one health disorder and the effects on the disease burden (e.g., characterized by the proportion of 11 health disorders involved) conditional on the subject developing the health disorder, respectively, and *f* (⋅) denotes a density function. Estimates for the parameters ($\alpha ,\beta ,\sigma^{2},\sigma_{u}^{2},\sigma_{v}^{2},\rho$) can be obtained by maximizing the above likelihood function. However, this optimization could be challenging since the likelihood function involves two intractable integrals, which usually cannot be dealt with separately, except in the case of independent data [22]. Several strategies can be adopted, for example, the Fisher score approach via Laplace approximation [21], or the quasi-Newton optimization via adaptive Gaussian quadrature [23, 24]. In this paper, we adopt a hybrid expectation maximization (EM) and quasi-Newton algorithm [25].

To test the *k*^*th*^ hypothesis: $H_{k}^{0}:\alpha_{k}=\beta_{k}=0$ vs $H_{k}^{1}\;:\;\text{otherwise}\;\left(k=1,\cdots ,p\right)$, we construct the following test statistic:

$$\begin{array}{c} \zeta =\left({\hat{\alpha }}_{k},{\hat{\beta }}_{k}\right)\Sigma^{-1}(\begin{array}{c} {\hat{\alpha }}_{k}\\ {\hat{\beta }}_{k} \end{array}) \end{array}$$ (4)

where ${\hat{\alpha }}_{k}$ and ${\hat{\beta }}_{k}$ are the maximum likelihood estimates (MLEs) for parameters *α*_*k*_ and *β*_*k*_, respectively, from Model (3). They follow a bivariate normal distribution with mean zero under the null as follows:

$$\begin{array}{c} \left(\begin{array}{c} {\hat{\alpha }}_{k}\\ {\hat{\beta }}_{k} \end{array})∼N((\begin{array}{c} 0\\ 0 \end{array}),\Sigma \right) \end{array}$$ (5)

where $\Sigma =(\begin{array}{cc} \sigma_{\alpha_{k}}^{2} & \varrho \sigma_{\alpha_{k}}\sigma_{\beta_{k}}\\ \varrho \sigma_{\alpha_{k}}\sigma_{\beta_{k}} & \sigma_{\beta_{k}}^{2} \end{array})$ is the covariance matrix between ${\hat{\alpha }}_{k}$ and ${\hat{\beta }}_{k}$, with $\sigma_{\alpha_{k}}^{2}$ and $\sigma_{\beta_{k}}^{2}$ being the variance of the MLEs, respectively, and *ϱ* being the correlation between these MLEs. Σ can be estimated by maximizing the likelihood function (3) as well. Test statistic *ζ* follows a *χ*^2^(2) distribution under the null.

In summary, the existing analysis method for this type of correlated semi-continuous PCHI data, i.e. Model (1), only models the relationship between the risk factors and the chance to be in positive health disorder status by dichotomizing PCHI scores (i.e., PCHI = 1 vs PCHI< 1). However, the risk factors usually not only influence the chance of being in health disorder status but also impact the disease burden. The proposed analysis method, i.e. Model (3), considers the risk factors effect on both the probability and burden of being in health disorder by incorporating Models (1) and (2). Furthermore, although this is a cross-sectional study (i.e. PCHI scores measured at age of 10-year), the proposed analysis method adopted mixed-effect models for longitudinal data to take into account the correlations between and within the twin’s and triplet’s PCHI (health disorder) measurements in the two data processes.

## 3 A practical application

To investigate the performance of the proposed method in practice, we apply it to the analysis of data from the Extremely Low Gestational Age Newborn (ELGAN) study, which has motivated this research. The ELGAN study is a prospective longitudinal observational cohort study. Study participants were enrolled at 14 hospitals in 5 states in the United States (Connecticut, Massachusetts, Illinois, Michigan, and North Carolina) between April 2002 and August 2004. The only inclusion criteria were birth at one of the enrollment hospitals and birth before 28 completed weeks of gestation [26, 27]. The only exclusion criterion was anencephaly. Longitudinal follow-up of the 1198 surviving participants in the ELGAN cohort has included research clinic visits at 1, 2, 10, and 15 years of age. Children were diagnosed with cerebral palsy based on a standardized assessment at 2 years of age, adjusted for degree of prematurity [28]. All other assessments that were used to derive the positive child health index occurred when study participants were 10 years of age. Parent report was used to identify bilateral blindness and hearing impairment. Children’s weight and height were measured by research coordinators at 10 years of age and body mass index (BMI) percentiles were calculated based on measured weight and height and age- and sex-specific US growth standards; obesity was defined as a BMI percentile ≥ 95% [29]. Asthma diagnosis at age 10 years was based on parent or guardian report of a health care provider’s diagnosis of asthma [30]. Here, we focus on estimating the impacts of sex, race, birth weight, maternal pre-pregnancy BMI, maternal smoking status during pregnancy, maternal age, maternal education level, histologic chorioamnionitis (a prenatal infection of the fetal membranes), pre-pregnancy maternal asthma status, and insurance status (public health insurance at birth) on the health disorders measured at age of 10 year-old for extremely preterm-born children, and identify the important factors associated with the health disorders. The data consist of 776 complete samples in total, of which 550 are singletons, 188 are twins (94 pairs), 33 are triplets (11 triplets), and 5 are quintuplets. The distributions of all observed clinical and demographic variables are summarized in Table 1. We note that nearly one third of the subjects (*n* = 251) reported no any health disorders at age 10.

**Table 1:** Distribution of child and maternal characteristics.

Child Characteristics		N or Mean	Percentage or SD
Number of disorder	None	251	32.3
	At least one	525	67.7
Sex	Female	369	47.6
	Male	407	52.4
Race	White	498	64.2
	Other	278	35.8
Birth weight		835.8	197.6
Maternal Characteristics		N or Mean	Percentage or SD
Smoking status during pregnancy	Yes	107	13.8
	No	669	86.2
Pre-pregnancy asthma	Yes	92	11.9
	No	684	88.1
Public insurance at birth	Yes	269	34.7
	No	507	65.3
Maternal education	≤ 12 years	103	13.3
	> 12 years	673	86.7
Histologic chorioamnionitis	Yes	268	34.5
	No	508	65.5
Pre-pregnancy BMI		25.5	6.9
Maternal age		29.3	6.7

We compare the performance of our proposed analysis method, i.e. the joint mixed-effects model (3) which jointly tests the effect of each feature using the test statistic (4) as described above, with the existing analysis method for PCHI data, i.e. mixed-effects logistic regression approach (1). In our analysis, pre-pregnancy asthma and histologic chorioamnionitis were included in the two-part model since they are potential confounding variables for the health disorders [12]. The parameter estimates and associated 95% confidence interval (CI) are presented in Table 2. As shown, at the 0.05 level of significance, the conventional approach detects sex, race, maternal pre-pregnancy BMI and public health insurance (rather than private insurance) as important risk factors, while the joint-test procedure identifies sex, birth weight, maternal pre-pregnancy BMI and public health insurance. However, after adjusting for multiple comparisons using the Hommel [31] or Bonferroni correction, only maternal pre-pregnancy BMI remains significant in the conventional method with an adjusted p-value of 0.008 (the adjusted p-values for sex, race and public health insurance become 0.177, 0.189, 0.115, respectively). Conversely, sex, maternal pre-pregnancy BMI and public health insurance remain significant in the joint-test procedure after this adjustment, with p-values of 0.010, 0.005, and 0.005, respectively. Although both methods detect pre-pregnancy BMI as a significant risk factor, we note that the adjusted p-value from the joint-test procedure is much smaller than that from the conventional method (0.005 vs 0.008). The consistency of corresponding pairs of parameter estimates from the mixed-effects two-part model is reassuring. That is, the estimates for sex (0.386;0.143), maternal pre-pregnancy BMI (0.346;0.053), and public health insurance (0.594;0.188), indicate that each influences the propensities of incurring health disorders and the health disorder burden (conditional on at least one health disorder) in the same direction. Even though the parameter estimate (i.e., *α*) difference between existing method and the proposed method is small, joint-modeling includes the information from the second model (i.e. *β*). For example, using existing method, birth weight effect estimate is −0.143, which is not statistically significant. However, using the proposed joint-modeling method, the birth weight effect on the propensity of developing health disorder is estimated as −0.157, yet the birth weight is detected as a significant factor (p-value = 0.023) since the joint-modeling method considers the birth weight effect on the disease burden with a parameter estimate of −0.052. This provides intuition to understand why the joint modeling can potentially boost detection power compared with the conventional method. Also, in our analysis, treating pre-pregnancy BMI as a continuous or categorical variable won’t change the overall conclusion.

**Table 2:** PCHI data analysis results.

	Existing Method		Model (2)		Proposed Method	
Variable	Estimate (*α*)	95% CI	Estimate (*β*)	95% CI	Estimate (*α*;*β*)	p-value
Sex	0.380	(0.047,0.713)	0.141	(0.049, 0.234)*	0.386; 0.143	0.001*
Race	0.451	(0.051,0.850)	0.036	(-0.068,0.140)	0.498; 0.040	0.067
Birth weight	-0.143	(-0.309,0.023)	-0.050	(-0.096,-0.005)	-0.157;-0.052	0.023
Pre-pregnancy BMI	0.330	(0.137,0.523)*	0.050	(0.005,0.094)	0.346; 0.053	0.001*
Smoking status during pregnancy	0.403	(-0.149,0.955)	0.076	(-0.057,0.209)	0.438; 0.080	0.188
Maternal age	-0.177	(-0.375,0.021)	0.021	(-0.034,0.077)	-0.183; 0.021	0.181
Maternal education	-0.178	(-0.785,0.430)	-0.119	(-0.258,0.019)	-0.173;-0.120	0.209
Histologic chorioamnionitis	0.109	(-0.244,0.462)	0.047	(-0.051,0.144)	0.133; 0.049	0.495
Pre-pregnancy asthma	0.373	(-0.200,0.946)	0.101	(-0.035,0.236)	0.403; 0.102	0.159
Public health insurance at birth	0.564	(0.104,1.025)	0.182	(0.068,0.296)*	0.594; 0.188	0.001*

*statistically significant at significance level 0.05 after adjusting for multiple comparison

## 4 Simulation study

The semi-continuous nature of the PCHI data is actually a relatively common feature of scientific and clinical data. For example, medical expenditures and length of hospital stay are two other examples that give rise to such data; in a given year some people accrue no medical expenses (or require no time in hospital) at all. Otherwise, these outcomes are represented by positive numbers (i.e., dollars or days). Examples of such data abound in economic studies—for example, the size of an insurance claim will be a positive number when an incident occurs, and zero otherwise. Other examples include postoperative pain (POP) measured sometime post surgery; many surgery patients experience POP with varying intensity (i.e., the POP scores take positive values), while POP will completely resolve by the measurement occasion for many others (i.e., the POP scores are zero). An example with particular relevance to studies of extremely preterm babies is the duration of mechanical ventilation, which is 0 for a substantial proportion of infants and continuous positive values for others.

We conducted extensive simulation studies to explore the applicability of the joint-test procedure under scenarios of varying data complexity. In our first scenario, we simulated the correlated data using a mixed-effects logistic regression model to generate the binary adverse health status *z*, and a log-normal linear mixed-effects model to generate the positive data process, i.e., the burden of the adverse health outcome *y*, as presented below:

$$\begin{array}{c} \begin{array}{cc} & \text{logit}\left(\pi_{ij}\right)=0.8+0.2x_{1,ij}+0.4w_{1,ij}+0.45x_{2,ij}+0.6w_{2,ij}+u_{i}\\ & \text{log}\left(y_{ij}\right)=-0.5+0.25x_{1,ij}+0.5w_{1,ij}+0.4x_{3,ij}+0.6w_{3,ij}+v_{i}+\epsilon_{ij}\;\text{if}\;z_{ij}=1 \end{array} \end{array}$$

where *π*_*ij*_ ≡ Pr(*z*_*ij*_ = 1|{*x*_1,*ij*_, *x*_2,*ij*_, *w*_1,*ij*_, *w*_2,*ij*_, *u*_*i*_}) and *ϵ*_*ij*_ ∼ *N*(0, 1) is a random error term. In addition to the causal risk factors (i.e. *x*_1_, *x*_2_, *x*_3_ and *w*_1_, *w*_2_, *w*_3_, which correspond to continuous and binary variables, respectively), we also generate two nuisance factors, one continuous *x*_4_ and the other binary *w*_4_. The continuous variables follow a multivariate normal distribution and the binary variables correlate with the continuous variables *x*_4_, ⋯, *x*_8_ as follows:

$$\left(\begin{array}{c} x_{1}\\ x_{2}\\ \vdots \\ x_{8} \end{array})∼N((\begin{array}{c} 0\\ 0\\ \vdots \\ 0 \end{array})\;,\;(\begin{array}{cccc} 1 & 0.3 & \cdots & 0.3\\ 0.3 & 1 & 0.3 & \cdots \\ \vdots & 0.3 & 1 & 0.3\\ 0.3 & \cdots & 0.3 & 1 \end{array})\right)$$

$$w_{p}=\{\begin{array}{cc} 1 & \text{if}\;x_{p+4}>0\\ 0 & \text{if}\;x_{p+4}\leq 0 \end{array}\;\;\;\;\;\;\;\;\;\;\left(p=1,\cdots ,4\right)$$

The random-effects terms *u*_*i*_ and *v*_*i*_ follow a bivariate normal distribution as follows:

$$(\begin{array}{c} u_{i}\\ v_{i} \end{array})∼N((\begin{array}{c} 0\\ 0 \end{array})\;,\;(\begin{array}{cc} 1 & \rho \\ \rho & 1 \end{array})).$$

Using sample sizes of 500 (400 singletons and 50 twin pairs) and 1000 (800 singletons and 100 twin pairs), we report the percentage of 1000 Monte Carlo replications run that identify each of the eight risk factors (*X*_1_, ⋯, *X*_4_, *W*_1_, ⋯, *W*_4_) as statistically significant under different correlation settings for (*ρ* = 0 and *ρ* = 0.4) in Tables 3 and 4, respectively, controlling the type I error rate at the 0.05 level.

**Table 3:** Power and type I error comparison for cluster log-normal data (*ρ* = 0).

	*n* = 50			*n* = 1000		
Variable	Existing Method	Model (2)	Proposed Method	Existing Method	Model (2)	Proposed Method
*X*_1_	0.298	0.400	0.859	0.509	0.597	0.993
*W*_1_	0.332	0.316	0.880	0.562	0.523	1.000
*X*_2_	0.880	0.053	0.729	0.995	0.046	0.988
*W*_2_	0.602	0.035	0.408	0.906	0.038	0.811
*X*_3_	0.051	0.754	0.993	0.055	0.926	1.000
*W*_3_	0.055	0.454	0.956	0.049	0.730	1.000
*X*_4_	0.056	0.059	0.048	0.044	0.052	0.037
*W*_4_	0.040	0.045	0.042	0.056	0.041	0.050

**Table 4:** Power and type I error comparison for cluster log-normal data (*ρ* = 0.4).

	*n* = 50			*n* = 1000		
Variable	Existing Method	Model (2)	Proposed Method	Existing Method	Model (2)	Proposed Method
*X*_1_	0.302	0.394	0.841	0.505	0.592	0.991
*W*_1_	0.319	0.302	0.867	0.569	0.515	0.993
*X*_2_	0.893	0.055	0.739	0.994	0.057	0.989
*W*_2_	0.609	0.044	0.414	0.903	0.037	0.813
*X*_3_	0.059	0.783	0.996	0.049	0.933	1.000
*W*_3_	0.059	0.452	0.940	0.053	0.740	1.000
*X*_4_	0.053	0.052	0.034	0.063	0.041	0.043
*W*_4_	0.055	0.039	0.045	0.046	0.036	0.046

The results in Tables 3 and 4 demonstrate that the joint-test procedure (i.e., the column labeled “Proposed Method”) outperforms the traditional mixed-effects logistic regression method (“Existing Method”) in detecting the important factors. For example, when *n* = 500 and *ρ* = 0, the power to detect the risk factors *X*_1_ and *W*_1_ (which influence both the binary events and the continuous positive outcomes) are markedly boosted from 0.298 and 0.332 to 0.859 and 0.880, respectively. Similar observations pertain to the other settings for varying *n* and *ρ*. Furthermore, when the traditional method fails to detect risk factors that only influence the continuous outcome, the joint-test procedure still can detect these factors with very high power. For instance, under the setting of *n* = 500 and *ρ* = 0.4, the traditional method fails to detect *X*_3_ and *W*_3_ (the negligible powers 0.059 and 0.059 are essentially equal to the nominal type I error rate), but the joint-test procedure has nearly full power to detect these two risk factors. In addition, the empirical type I error is appropriately controlled.

To further investigate the robustness of our two-part modeling approach to identify important biomarkers for correlated data, we conducted additional simulations using the following data generating models:

$$\begin{array}{ccc} & & \text{logit}\left(\pi_{ij}\right)=0.8+0.25x_{1,ij}+0.2w_{1,ij}+0.4x_{2,ij}+0.6w_{2,ij}+u_{i}\\ & & y_{ij}∼\text{Poisson}\left(16+0.65x_{1,ij}+0.85w_{1,ij}+0.5x_{3,ij}+w_{3,ij}+v_{i}\right)\;\text{if}\;z_{ij}=1 \end{array}$$

where the causal risk factors, nuisance variables, and cluster effects settings are the same as in the above cluster log-normal scenario. Here, we still employ a multiple linear log-normal mixed-effects model for the positive portion of the data, but these data are now generated using a Poisson mixed-effects model. We ran 1000 Monte Carlo replications with a sample size *n* = 1000 and two different levels of correlation between the cluster effects (*ρ* = 0 and *ρ* = 0.4). The percentage of replications deeming the eight risk factors as statistically significant (while controlling the type I error rate at the 0.05) level are presented in Table 5. Again, the results in Table 5 demonstrate that the joint-test procedure under two-part modeling (“Proposed Method”) is much more powerful than the traditional one-part mixed-effects logistic regression model (“Existing Method”) to detect risk factors while controlling the type I error rate at a reasonable level regardless of the correlation settings. Of note, even when the intensity model (i.e., the log-normal mixed-effects model) is misspecified for the positive ordinal data in the two-part modeling framework, the joint-test procedure still outperforms the traditional approach, further demonstrating its utility in clinical practice.

**Table 5:** Power and type I error comparison for cluster poisson data.

	*ρ* = 0			*ρ* = 0.4		
Variable	Existing Method	Model (2)	Proposed Method	Existing Method	Model (2)	Proposed Method
*X*_1_	0.743	0.945	0.982	0.736	0.927	0.976
*W*_1_	0.192	0.688	0.654	0.183	0.703	0.656
*X*_2_	0.982	0.054	0.953	0.977	0.052	0.950
*W*_2_	0.884	0.047	0.810	0.893	0.050	0.805
*X*_3_	0.056	0.785	0.688	0.054	0.775	0.695
*W*_3_	0.048	0.861	0.756	0.057	0.845	0.752
*X*_4_	0.058	0.052	0.037	0.056	0.047	0.050
*W*_4_	0.043	0.057	0.045	0.053	0.047	0.045

## 5 Discussion

In this study, we derived a joint-test procedure under a mixed-effects two-part modeling framework to identify important risk factors associated with the correlated semi-continuous outcomes. The application of the proposed method to the real PCHI data analysis has clearly demonstrated the advantages of the proposed method compared to the existing method for this type of semi-continuous data, i.e., it is more powerful to detect risk factors associated with the correlated semi-continuous outcomes. Further, this method is robust to model misspecification. In general, our proposed joint-test procedure is consistently more powerful than the traditional method while controlling the type I error rate at the same targeted level. The advantages of the proposed two-part model rooted from the fact that it jointly models the two data processes unlike the existing method (e.g., the censored or truncated regression) assumes one underlying data process for all subjects with a ceiling effect and thus is less flexible. These results manifestly demonstrate the advantages of the mixed-effects two-part model for the analysis of correlated semi-continuous data.

In pediatric practice, in addition to identifying important risk factors associated with cross-sectional adverse health outcomes for preterm-born children, it is of clinical importance to investigate their associations with longitudinal health outcomes as well. It is of further practical importance to identify genetic variants that are associated with the health outcomes of these children. How to extend the proposed joint-test procedure to these even more complicated longitudinal correlated semi-continuous (and, in the latter case, high dimensional) settings warrants further investigations, but is beyond the scope of this paper.

## Supporting information

S1 Data (CSV)
